# Causal effect of serum 25-hydroxyvitamin D levels on low back pain: A two-sample mendelian randomization study

**DOI:** 10.3389/fgene.2022.1001265

**Published:** 2022-09-19

**Authors:** Xiaojuan Jiang, Ruihao Zhou, Yi He, Tao Zhu, Weiyi Zhang

**Affiliations:** ^1^ Department of Anesthesiology, National Clinical Research Center for Geriatrics, West China Hospital, Sichuan University, Chengdu, China; ^2^ The Research Units of West China (2018RU012)-Chinese Academy of Medical Sciences, West China Hospital, Sichuan University, Chengdu, China

**Keywords:** 25-hydroxyvitamin D, the causal effect, genome-wide association study, mendelian randomization, single nucleotide polymorphism, low back pain

## Abstract

**Background:** Previous observational studies have suggested the involvement of 25-hydroxyvitamin D [25(OH)D] in chronic pain. However, whether the 25(OH)D is a novel target for management, the causality remains unclear.

**Methods:** A two-sample Mendelian randomization (MR) study was conducted to identify the causal association between 25(OH)D and low back pain (LBP). The primary analysis was revealing causality from serum 25(OH)D level (*n* = 417,580) on LBP (21,140 cases and 227,388 controls). The replicated analysis was performing MR estimates from circulating 25(OH)D concentration (*n* = 79,366) on LBP experienced last month (118,471 cases and 343,386 controls). Inverse variance weighted (IVW) was used as the main analysis. In addition, we used weighted median and MR-Egger to enhance the robustness. Sensitivity analysis was conducted to evaluate the robustness of MR results.

**Results:** IVW estimation indicated strong evidence that higher serum 25(OH)D levels exerted a protective effect on LBP (OR = 0.89, 95% CI = 0.83–0.96, *p* = 0.002). Similar trends were also found in replicate analysis (OR = 0.98, 95% CI = 0.96–1.00, *p* = 0.07). After meta-analysis combining primary and replicated analysis, the causal effect is significant (*p* = 0.03). Sensitivity analysis supported that the MR estimates were robust.

**Conclusion:** In our MR study, genetically increased serum 25(OH)D levels were associated with a reduced risk of LBP in the European population. This might have an implication for clinicians that vitamin D supplements might be effective for patients with LBP in clinical practice.

## Introduction

Low back pain (LBP) is commonly regarded as a symptom rather than a disease. It has been the leading cause of loss from work and the main indication for medical rehabilitation both in developed and developing countries (Chenot et al., 2017; Maher et al., 2017). Through the systematic analysis of the GBD (Global Burden of Disease) study 2019, LBP ranked stably in the top ten over the past 30 years and affected people of all ages (Vos et al., 2020). Besides, the prevalence of LBP increased with age, ranging from 16.9% to 46.6% (Maher et al., 2017). Because of the unknown pathoanatomical cause, the treatment focuses on identifying the risk and protective factors to relieve pain.

Vitamin D deficiency is a major world pandemic. It has been linked to many human diseases such as Alzheimer’s disease (AD), Parkinson’s disease (PD), multiple sclerosis (MS), hypertension, and cardiovascular diseases (Berridge, 2015). Vitamin D status, as measured by 25-hydroxyvitamin D [25(OH)D], could potentially be involved in the pathophysiology of nonspecific pain (Hossein-nezhad and Holick, 2013). Also, there is increasing evidence from observational studies and some meta-analyses that 25(OH)D deficiency is associated with a wide range of chronic pain disorders (Hossein-nezhad and Holick, 2013; Wu et al., 2018). However, there is no consensus on the association between circulating 25(OH)D concentrations and pain from existing research. One meta-analysis consisting of seven observational studies with 2,420 statin-treated patients found that 25(OH)D levels were lower in those with myalgia than in those without (Michalska-Kasiczak et al., 2015). Another meta-analysis comprising 1854 participants found no difference of 25(OH)D levels between people with and without chronic widespread pain (Hsiao et al., 2015). Given the limited evidence and inconsistent conclusions from previous studies, the putative causal link between serum 25(OH)D levels and LBP remains unclear and further convincing evidence needs to be explored.

Mendelian randomization (MR), as an emerging method, is applied to determine the potential causal relationship between exposure factors and outcomes. Specific single-nucleotide polymorphisms (SNPs) were used as instrumental variables (IVs) (Emdin et al., 2017). Due to the random distribution of alleles during gamete formation, this design is less likely to be confounded or influenced by reverse causality. Based on the advantages of the study design, MR can well reveal causal effects from exposure and outcome. And with the booming of publicly available large sample size GWAS data, it is more efficient to obtain high statistic power. Previously, Mendelian randomization design has been used to explore the causal association of serum 25(OH)D with many diseases, such as major depression, diabetes, and multiple sclerosis (Afzal et al., 2014; Michaelsson et al., 2018; Wang, 2022), but it has not yet been applied to investigate its effect on LBP. Therefore, we conducted a two-sample MR study to investigate the causal effect between serum 25(OH)D levels and LBP.

## Materials and methods

### Study design

This MR study is based on the large-scale GWAS summary datasets. All participants have given informed consent in all these corresponding original studies and additional ethics approval was not needed as we only used summary-level statistics. The primary analysis was based on the serum 25(OH)D concentration (*n* = 417,580) from the IEU consortium on LBP from the FinnGen consortium (*n* = 248,528). Then, we performed a replicated MR analysis with GWAS data of circulating 25(OH)D concentrations from a GWAS of 79,366 participants on LBP experienced last month from the IEU consortium (*n* = 461,857). Sensitivity analysis includes Cochran’s Q test, leave-one-out analysis, funnel plots, and MR-Egger intercept analysis. Radial MR and MR-Pleiotropy Residual Sum and Outlier method (MR-PRESSO) were used to detect outliers if heterogeneity or pleiotropy existed. Then multiplicate random effect IVW was performed to detect heterogeneity after removing outliers and calculating the causal effect.

As presented in Supplementary Figure S1, the two-sample MR study must meet three principal assumptions. According to assumption 1, genetic instrument variants are closely related to the level of serum 25(OH)D (exposure factor). Because SNP was randomly assigned during pregnancy, genetic instrument variants of 25(OH)D should not be confounded by any other factors based on assumption 2. Besides, we performed IVW analysis to evaluate whether causal effects exist between 25(OH)D on LBP potential risk factors, including alcohol intake frequency, BMI, and obesity. Assumption three was that the risk of outcome (LBP) was strongly influenced by 25(OH)D genetic instrument variants through the exposure factor [25(OH)D] but not through other pathways (Didelez and Sheehan, 2007). As previously described, the second and third assumptions are collectively known as independence from pleiotropy (Emdin et al., 2017).

### Genetic variants associated with serum 25(OH)D

The primary genetic instruments were derived from a recent GWAS dataset of serum 25(OH)D levels from the IEU consortium, comprising 417,580 individuals of European ancestry (Hemani et al., 2018). Typically, genome-wide significant genetic variants (*p* < 5 × 10^–8^) are selected as the potential instruments. To further obtain SNPs independent of each other, we then pruned these instruments within a window size of 10,000 kb to mitigate linkage disequilibrium (LD) at a threshold of r^2^ < 0.001. For SNPs, the F statistic was used to evaluate the strength of the correlation between instrumental variables and exposure factors. Only when the F statistic >10, it was considered that no bias was caused by weak instrumental variables (Pierce et al., 2011).

### GWAS summary data for low back pain

The GWAS summary data for LBP was obtained from the FinnGen consortium (round 7), which is a large public-private partnership consisting of 500,000 Finnish biobank participants. The genetic information for LBP was generated from 21,140 LBP cases and 227,388 controls. More details for the endpoint definition were reported at the website: https://risteys.finngen.fi/endpoint/M13_LOWBACKPAIN (Kurki et al., 2022).

### Replicated analysis of causal effect between 25(OH)D on low back pain

To detect the robustness of our MR estimates, we also used another GWAS dataset (*n* = 79,366, European ancestry) to represent circulating 25(OH)D concentration. In that study, Xia Jiang identified six SNPs significantly associated with circulating 25(OH)D concentration. The summary data of these six instrument variables was presented in Supplementary Table S2. More detail for the meta-analysis can be found in the original research (Jiang et al., 2018). The outcome was selected from the IEU consortium with a GWAS ID as “ukb-b-9838”, which represented the phenotype “back pain experienced in last month”. The number of cases is 118,471 and the number of controls is 343,386. In addition, we performed a meta-analysis with Revman (Version 5.4) to combine the primary and replicated MR analysis.

### Positive validation of the mendelian randomization analysis

To detect the robustness of the MR analysis method and the instrument variables used in our study, we performed the MR analysis from two sets of instrumental variables for 25(OH)D aforementioned on Vitamin D deficiency of FinnGen consortium. Brief information about the GWAS data utilized in the current study was listed in Supplementary Table S3. The presented traits of the used GWAS database included GWAS-ID, the ancestry, consortium, the number of cases and controls and the gender.

### Statistical analyses

The exposure SNPs were extracted from the full GWAS data of LBP. Harmonization was then processed to make the effect alleles of the exposure and outcome SNPs coincide, and rule out SNPs with incompatible alleles or being palindromic with intermediate effect allele frequency. To be more exact, there were four steps. First, we clump the SNPs to obtain independent genetic instrument variables. Second, proxied SNPs would be found for missed SNPs. Third, we discarded SNPs significantly associated with outcomes. Forth, ambiguous and palindromic SNPs were discarded. Then, MR analysis was conducted. Specifically, the inverse variance weighted (IVW) estimate was used as the primary MR effect estimate, reported as odds ratios (OR) and 95% confidence intervals (95% CI) (Burgess et al., 2013). We also estimated the causal effects using two other methods: the weighted median and MR-Egger regression methods. Those three approaches are considered as the most scientific and commonly used methods to provide robust analysis of the findings for a Mendelian randomization investigation (Chen et al., 2021; Chen et al., 2022). If the weighted median method is to be applied, at least 50% of SNPs have to satisfy the premise that they are valid instrumental variables (Bowden et al., 2016). The adaption of MR-Egger can detect some violations of the standard instrumental variable assumptions, and provide an effect estimate which is not subject to these violations (Ong and MacGregor, 2019).

Sensitivity analysis is a necessary method to evaluate the potential bias in Mendelian randomization studies. It includes the following two considerations, heterogeneity test, and pleiotropy test. The Cochran’s Q test was used to detect heterogeneity in the IVW approach, and the intercept from the MR-Egger regression indicated horizontal pleiotropy (intercept with *p* < 0.05 was considered as the presence of horizontal pleiotropy) (Bowden et al., 2015; Burgess and Thompson, 2017). Additionally, we removed SNPs with pleiotropic outliers using the MR-PRESSO and Radial MR (*p* < 0.05) (Bowden et al., 2018; Ong and MacGregor, 2019). When potential outliers were identified, we discarded them and repeated the IVW estimate to evaluate the robustness of our results. Besides, multiplicative random effect IVW was also performed. A leave-one-out analysis was also conducted to determine if the MR estimate was driven or biased by a single SNP. As the name suggested, SNPs were discarded one by one and then MR were reperformed, therefore can evaluate whether the causal estimate was drived by single SNP.

MR analysis was performed by R software (version 4.2.0), TwoSample MR package (version 0.5. 6), and RadialMR package (version 1.0). The main code utilized in our study was presented in https://github.com/XiaojuanJiang/LBP-analysis.

## Results

### Primary analyses

We successfully extracted 109 corresponding serum 25(OH)D-associated genetic variants from the LBP GWAS dataset. Among them, two SNPs (rs11606 and rs2246832) were removed for being palindromic in the harmonization process. Thus, 107 SNPs were included in the MR analysis. In our study, the F-statistics range from 23.8 to 6,559.1, larger than the conventional value of 10, suggesting that the instruments had a strong potential to predict 25(OH)D levels.

**FIGURE 1:**
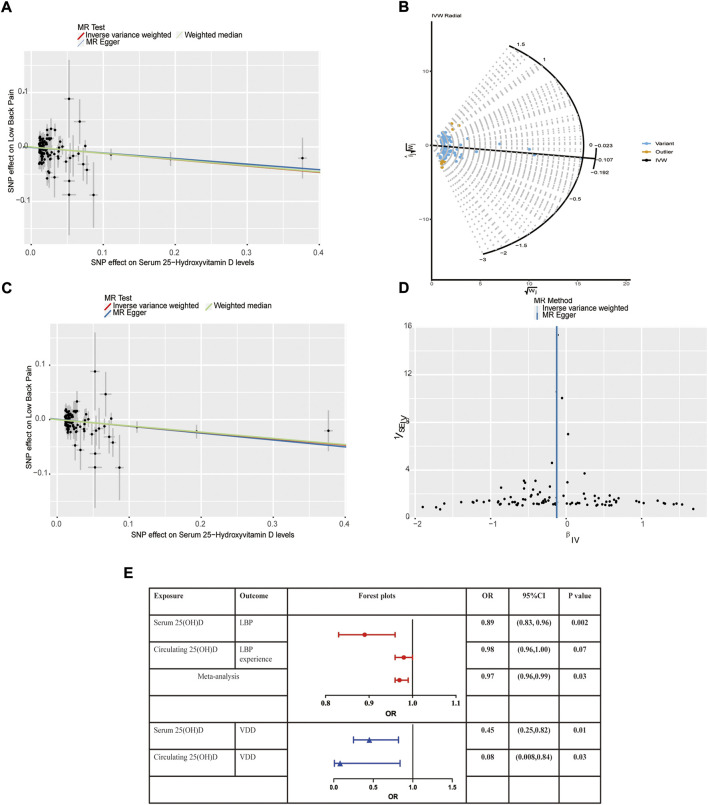
Mendelian randomization estimates from 25(OH)D on low back pain. **(A)** Scatter plot showing the causality of serum 25(OH)D on low back pain previous to removing outliers identified by MR-PRESSO and MR Radial; **(B)** Outliers identified by MR Radial; **(C)** Scatter plot showing the causality of serum 25(OH)D on low back pain after removing outliers identified by MR-PRESSO and MR Radial. **(D)** Funnel plots; **(E)** Forest plots of the IVW estimates.

We assessed the causal effect between serum 25(OH)D levels and LBP by using IVW, MR-Egger regression, and the weighted median method. The result of IVW indicated strong evidence that higher serum 25(OH)D levels had a causal effect on a decreased risk of LBP (OR = 0.89, 95% CI = 0.81–0.97, *p* = 0.01). Meanwhile, similar risk estimates were gained using the MR-Egger regression (OR = 0.90, 95% CI = 0.80–1.01, *p* = 0.09) and weighted median approaches (OR = 0.89, 95% CI = 0.80–0.99, *p* = 0.03). The concordance of the 3 MR models enhanced the reliability of a protective role of serum 25(OH)D level in the issue of LBP (Figure 1A). To comprehensively detect any potential bias in our MR study, sensitivity analysis using complementary methods was conducted. There was evidence of heterogeneity detected from the Cochran Q test (Q value = 163.92, *p* < 0.001). However, there was no evidence for a significant intercept (intercept = -0.0007, *p* = 0.728), indicating that there was no pleiotropy observed. Summary results of the pleiotropy test and heterogeneity test are shown in Table 1. Further, we conducted the MR-PRESSO and Radial MR analysis. One outlier (rs429358) was identified with MR-PRESSO and nine outliers (rs12056768, rs1260326, rs1800588, rs2346264, rs2659007, rs34284484, rs6724965, rs72834856 and rs7569755) were detected with Radial MR (Figure 1B). We manually removed this outlier and repeated IVW analysis, and the causality remained (OR = 0.89, 95% CI = 0.83–0.96, *p* = 0.002, Figure 1C). Details of the remained SNPs were listed in Supplementary Table S1. Multiple random effect IVW show a consistent and significant estimate (OR = 0.89, 95% CI = 0.83–0.96, *p* = 0.001) without heterogeneity (*p* = 0.61). Leave-one-out analysis was used to verify the impact of each SNP on the overall causal estimate. As shown in Supplementary Figure S2, when a certain SNP was excluded, the meta effect of the remaining SNPS was not across 0, indicating that the result did not change and reliable. The funnel plot was presented in Figure 1D. To be concluded, the causal effect from serum 25(OH)D level on LBP was not violated.

**TABLE 1:** Sensitivity analysis of serum 25(OH)D genetic IVs in GWAS for low back pain.

	Pleiotropy test			Heterogeneity test					
MR_egger			PRESSO	MR_egger			IVW		
Intercept	SE	*p*	*p*	Q	Q_df	Q_pval	Q	Q_df	Q_pval
−0.0007	0.002	0.728	<0.001	163.92	105	< 0.001	164.11	106	< 0.001

MR, mendelian randomization; SE, standard error. *p* ≥ 0.05 represents no significant pleiotropy. Q_pval ≥0.05 represents no significant heterogeneity. IVW, inverse variance weighted.

### Replicated analyses and meta-analysis

In the replicative analysis, MR estimates between circulating 25(OH)D and LBP showed a consistent but marginal significant result. IVW indicating that a higher circulating 25(OH)D concentration leads to a decreased risk of LBP (OR = 0.98, 95% CI = 0.96–1.00, *p* = 0.07). MR Egger and weighted median also showed a consistent but insignificant result (Table 2). No heterogeneity presents (*p* = 0.76) and no horizontal pleiotropy indicated (*p* = 0.35). After combining the primary and replicated MR analyses, the significant protective effect of 25(OH)D concentration on LBP remained (OR 0.97, 95% CI 0.96–0.99, *p* = 0.03, Figure 1E).

**TABLE 2:** Mendelian randomization from circulating 25(OH)D concentration on low back pain experienced last month.

MR methods	OR	95% CI	*p*	MR-egger intercept *p* value	Cochran’s Q test *p* value
IVW	0.98	0.96–1.00	0.07	0.76	0.35
MR Egger	1.00	0.97–1.03	0.89		
Weighted median	0.98	0.97–1.01	0.18		

MR, mendelian randomization; IVW, inverse variance weighted; OR, odds ratio.

### Potential risk factors analysis and positive control analyses

The IVW estimates of the primary and replicated instrument variables on the LBP potential risk factors were shown in Table 3. No evidence of causal effects existed, suggesting causal effect from 25(OH)D on LBP was not biased.

**TABLE 3:** The IVW estimates of the primary and replicated instrument variables on the LBP potential risk factors.

Exposure	Outcome	OR/beta	95% CI	*p*
Serum 25(OH)D concentration	Alcohol intake frequency	−0.037	−0.085 to 0.11	0.13
	BMI	0.011	−0.025 to 0.048	0.53
	Obesity	1.00	0.998 to 1.001	0.54

Theoretically, a higher 25(OH)D concentration would lead to a lower risk of vitamin D deficiency. In our positive control analyses, both higher serum 25(OH)D concentration and circulating 25(OH)D concentration causally decrease the risk of vitamin D deficiency (Table 4). No heterogeneity or pleiotropy was detected in the positive control analyses.

**TABLE 4:** Inverse variance weighted estimates for positive control analyses.

Exposure	OR	95% CI	*p*	MR-egger intercept *p* value	Cochran’s Q test *p* value
Serum 25(OH)D concentration	0.45	0.25–0.82	0.01	0.71	0.06
Circulating 25(OH)D concentration	0.08	0.008–0.84	0.03	0.33	0.14

## Discussion

This is the first study using the two-sample MR method to explore the causal association between serum 25(OH)D and LBP based on large-scale GWAS data. Briefly, our results strongly indicated that higher serum 25(OH)D levels had a causal effect on a decreased risk of LBP through the main estimation of IVW (OR = 0.89, 95% CI = 0.83–0.96, *p* = 0.002).

LBP, as one of the non-specific consequences of musculoskeletal diseases, has major effects on physical health by limiting mobility and quality of life. And LBP contributed to a serious burden on individuals and the medical care system. Because of the unclear pathophysiological cause, no specific treatments can be provided. In several studies and different populations, Vitamin D deficiency has been suggested as a possible contributing factor in the pathogenesis and maintenance of LBP (Johansen et al., 2013; Ghai et al., 2015; Thorneby et al., 2016; Wu et al., 2019). Some studies support the associations. Silva et al. compared 9,305 women with and without hypovitaminosis D and showed a higher frequency and severity of back pain in the former (69.5% vs. 66.9%, *p* = 0.022) (e Silva et al., 2013). While other studies failed to demonstrate any relationships between them. In a nested case-control, prospective study of the Norwegian community, no association between vitamin D status and risk of chronic LBP was found in the total data set (OR = 1.01, 95% CI 0.97–1.06) or in individuals with blood samples (OR = 0.99, 95% CI 0.93–1.06) (Heuch et al., 2017). Also, a double-blind trial on the role of vitamin D in nonspecific LBP showed no significant improvement in pain when compared with those given a placebo (Sandoughi et al., 2015). Another cross-sectional case-control study demonstrated no difference in vitamin D levels between participants with LBP and matched controls (Thorneby et al., 2016). These inconsistent conclusions may stem from many confounding reasons, such as various study designs and different populations. In our study, we used the method of Mendelian randomization analysis. In this approach, genetic instruments can be used to infer causal relationships in a potential causal manner, avoiding bias due to confounding, and estimating the putative causal relationship under different conditions. Besides, we replicate the analysis in another independent GWAS dataset and performed a meta-analysis. The potential risk factors and a series of sensitivity analyses were also performed to avoid violations. Thus, our results provided a robust conclusion that serum 25(OH)D has a causal effect on LBP.

To date, there is still no conclusive mechanism for how Vitamin D affects LBP. Several mechanisms have been proposed to link Vitamin D to the pathogenesis of LBP. Firstly, Vitamin D is a steroid hormone and plays a crucial role in the maintenance of bone and calcium homeostasis. As a result of reduced serum 25(OH)D concentrations, bone mass declines and bone pain develops (Heath and Elovic, 2006). Secondly, vitamin D receptors (VDR) are widely expressed in many tissues. Underlying the potential physiological actions for vitamin D, genetic deletion of this receptor can lead to poor muscle function (Boland, 2011). It has been ascertained that vitamin D deficiency increases the risk of falling, low muscle strength, and balance disorders (Saponaro et al., 2020). Another pathophysiologic explanation is the relationship of inflammation with vitamin D deficiency. As a result of vitamin D modulation of the RANK-RANKL osteoprotegerin system, an inflammatory cytokine response is triggered (Lacativa and Farias, 2010). Besides, other mechanisms have been proposed to link vitamin D deficiency to chronic pain, including the effects of immunoregulatory and proinflammatory, central and peripheral pain regulation of cytokine, and muscular effects of secondary hyperparathyroidism (Haroon and FitzGerald, 2012).

To the best of our knowledge, this is the first MR to explore the causal relationship between serum 25(OH)D and LBP. Our study has several major strengths. Firstly, GWAS datasets for LBP and serum 25(OH)D genetic IVs are both derived from European people, which avoids the effects of population stratification. Secondly, the selected GWAS datasets included a large sample size that contains millions of SNPs detected, largely improving the statistical power. Most importantly, to ensure the stability of the results, we conducted a replicate analysis and several additional methods to support the robustness of the estimation. As our findings suggested, supplementing vitamin D might relieve patients’ sufferings from LBP. And those with LBP symptoms could detect their serum 25(OH)D concentration to exclude vitamin D deficiency-dependent LBP.

Limitations should also be noted in this MR. Firstly, we identified the assumed causal effect between 25(OH)D and LBP in European ancestries. Future studies were necessary to extend our conclusion to other populations. Secondly, the mechanisms by which genetically increased serum 25(OH)D reduce the risk of LBP in the European population need to be further verified. Thirdly, we only used summary-level statistics, and hence stratification analysis was not allowed.

## Conclusions

In our MR study, genetically increased serum 25(OH)D levels were associated with reduced risk of LBP in the European population. This might have an implication for clinicians that vitamin D supplements might be effective for patients with LBP in clinical practice.

## Data Availability

The original contributions presented in the study are included in the article/Supplementary Material, further inquiries can be directed to the corresponding author.

## Supplementary material

Supplementary Figure S1 Three basic assumptions of the two-sample Mendelian randomization study design.

Supplementary Figure S2 The leave one out plot indicating the causal effect is not violated by single SNP.
