# Supplementary figures and images for: Causal effect of serum 25-hydroxyvitamin D levels on low back pain: A two-sample mendelian randomization study
Source: Front Genet. 2022 Sep 19;13:1001265. doi: 10.3389/fgene.2022.1001265 (PMC9534573; doi:10.3389/fgene.2022.1001265)

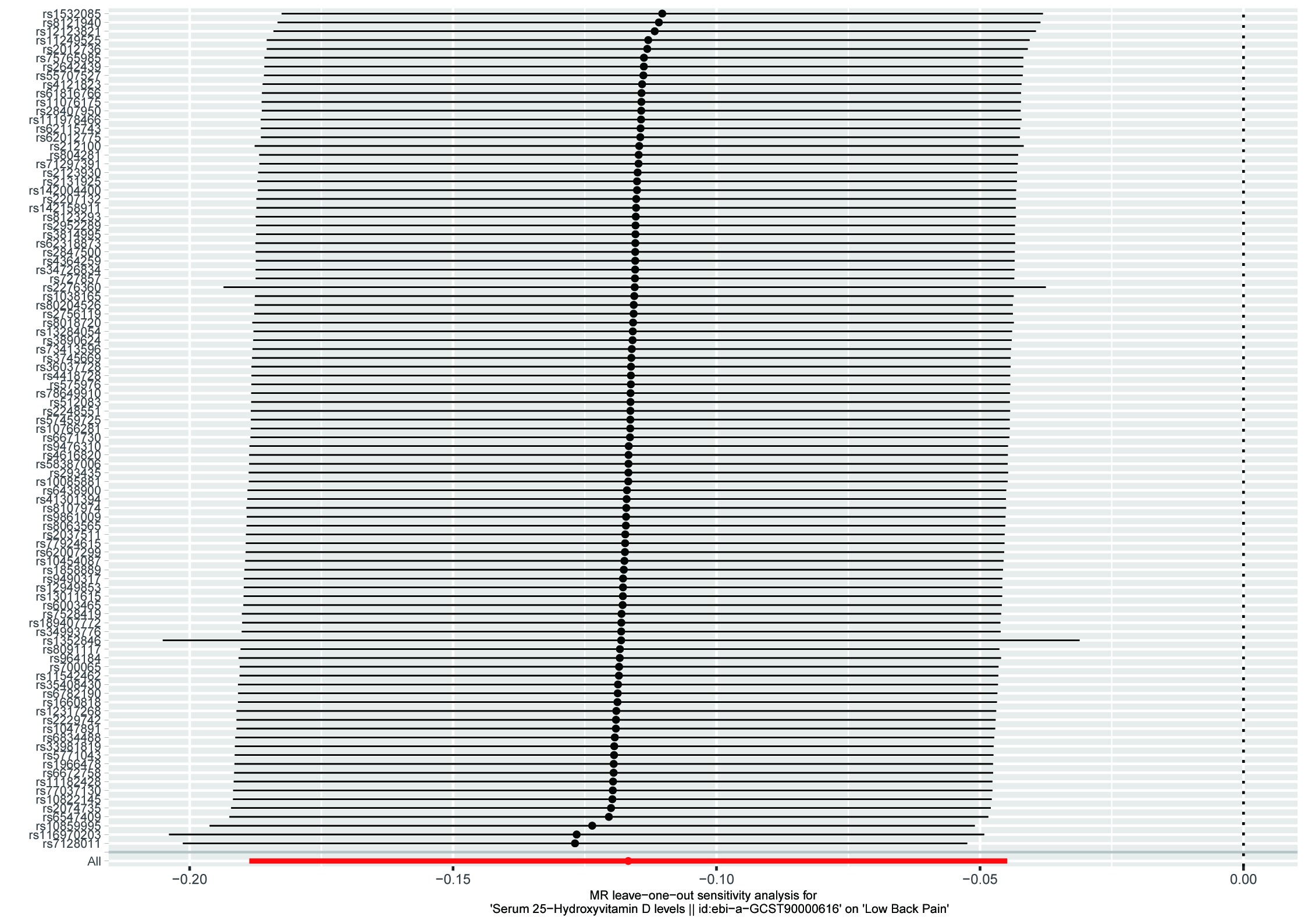

Supplement: Supplementary file 1 [file Image2.TIF]

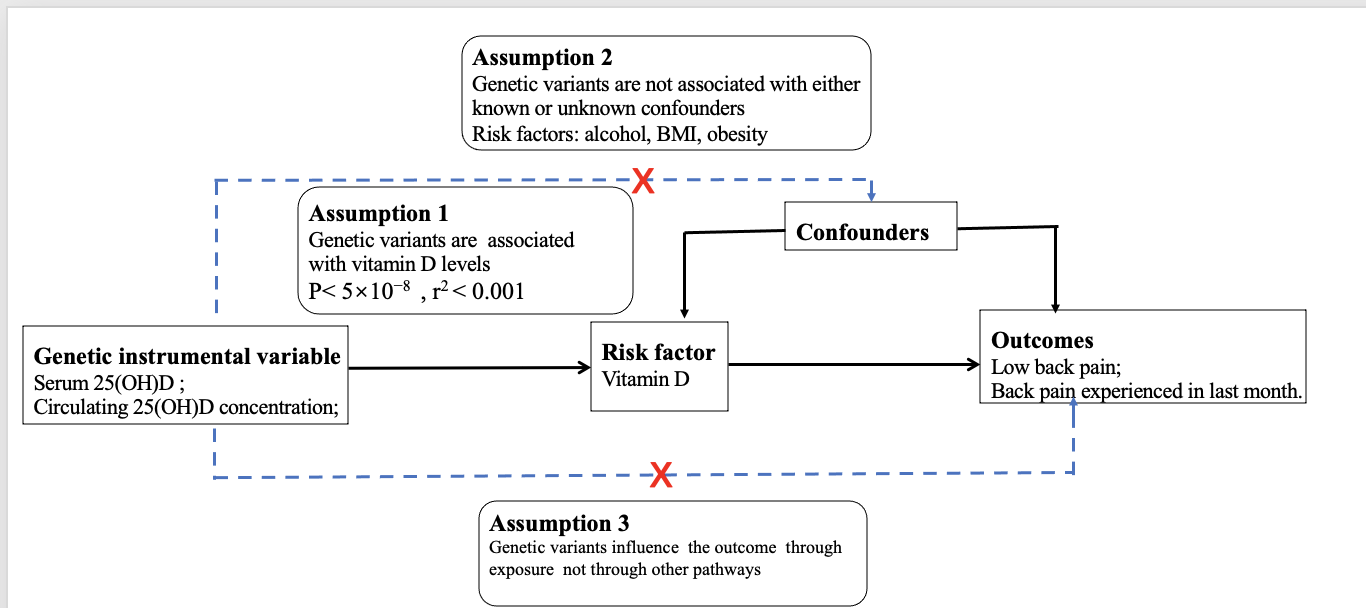

Supplement: Supplementary file 2 [file Image1.TIF]
