# Supplementary material for: Causal effect of serum 25-hydroxyvitamin D levels on low back pain: A two-sample mendelian randomization study
Source: Front Genet. 2022 Sep 19;13:1001265. doi: 10.3389/fgene.2022.1001265 (PMC9534573; doi:10.3389/fgene.2022.1001265)
Supplement: Supplementary file 3 [file DataSheet1.docx]

**Table S1** Details of the instrument variables for serum 25(OH)D level included in MR analysis.

| **SNP** | **effect_allele** | **other_allele** | **beta** | **eaf** | **se** | **p val** |
| --- | --- | --- | --- | --- | --- | --- |
| rs10085881 | C | T | -0.015 | 0.282 | 0.002 | 2.17E-11 |
| rs1038165 | T | C | 0.012 | 0.583 | 0.002 | 1.84E-09 |
| rs10454087 | T | C | -0.012 | 0.285 | 0.002 | 1.90E-08 |
| rs1047891 | A | C | -0.014 | 0.316 | 0.002 | 1.38E-10 |
| rs10766281 | G | A | 0.015 | 0.256 | 0.002 | 7.46E-12 |
| rs10822145 | T | C | -0.012 | 0.475 | 0.002 | 5.71E-10 |
| rs10859995 | C | T | -0.040 | 0.583 | 0.002 | 8.90E-91 |
| rs11076175 | G | A | 0.024 | 0.178 | 0.003 | 5.10E-20 |
| rs11182428 | C | T | -0.013 | 0.520 | 0.002 | 1.22E-10 |
| rs111978466 | T | C | 0.072 | 0.017 | 0.008 | 4.48E-21 |
| rs11249525 | T | C | -0.032 | 0.455 | 0.002 | 1.10E-59 |
| rs11542462 | A | G | -0.023 | 0.134 | 0.003 | 8.70E-16 |
| rs116970203 | A | G | -0.377 | 0.027 | 0.006 | 1.00E-200 |
| rs12123821 | T | C | 0.077 | 0.048 | 0.005 | 2.87E-63 |
| rs12317268 | G | A | -0.021 | 0.151 | 0.003 | 1.91E-14 |
| rs12949853 | A | G | 0.014 | 0.807 | 0.003 | 2.92E-08 |
| rs13284054 | C | T | 0.018 | 0.118 | 0.003 | 4.60E-09 |
| rs1352846 | G | A | -0.194 | 0.291 | 0.002 | 1.00E-200 |
| rs142004400 | C | A | -0.032 | 0.035 | 0.005 | 1.83E-09 |
| rs142158911 | A | G | 0.026 | 0.115 | 0.003 | 2.55E-17 |
| rs143069752 | A | T | 0.022 | 0.067 | 0.004 | 3.39E-08 |
| rs1532085 | G | A | 0.026 | 0.615 | 0.002 | 1.25E-38 |
| rs1660818 | A | G | 0.016 | 0.323 | 0.002 | 3.65E-14 |
| rs1858889 | C | A | 0.011 | 0.502 | 0.002 | 1.28E-08 |
| rs189407772 | G | A | 0.053 | 0.023 | 0.007 | 2.03E-15 |
| rs1966478 | C | T | -0.012 | 0.693 | 0.002 | 1.09E-08 |
| rs2012736 | A | C | -0.048 | 0.081 | 0.004 | 1.14E-40 |
| rs2037511 | A | G | 0.017 | 0.166 | 0.003 | 4.85E-11 |
| rs2074735 | C | G | 0.027 | 0.064 | 0.004 | 7.89E-12 |
| rs212100 | C | T | -0.066 | 0.836 | 0.003 | 1.38E-135 |
| rs2123930 | A | G | -0.014 | 0.279 | 0.002 | 9.14E-11 |
| rs2131925 | T | G | -0.022 | 0.644 | 0.002 | 5.84E-27 |
| rs2207132 | A | G | -0.037 | 0.033 | 0.005 | 1.34E-11 |
| rs2229742 | C | G | -0.025 | 0.103 | 0.003 | 1.12E-14 |
| rs2248551 | A | G | -0.021 | 0.165 | 0.003 | 6.67E-16 |
| rs2276360 | C | G | 0.111 | 0.788 | 0.002 | 1.00E-200 |
| rs2642439 | G | A | -0.015 | 0.685 | 0.002 | 1.47E-12 |
| rs2756119 | A | G | 0.013 | 0.383 | 0.002 | 4.25E-10 |
| rs28407950 | T | C | -0.014 | 0.244 | 0.002 | 9.41E-10 |
| rs2847500 | A | G | -0.022 | 0.124 | 0.003 | 1.15E-13 |
| rs293435 | T | C | -0.018 | 0.286 | 0.002 | 5.10E-16 |
| rs2952289 | T | C | 0.018 | 0.798 | 0.002 | 7.49E-13 |
| rs33981819 | G | T | 0.012 | 0.461 | 0.002 | 4.09E-09 |
| rs34726834 | T | C | 0.015 | 0.252 | 0.002 | 8.24E-11 |
| rs34993776 | T | C | 0.016 | 0.199 | 0.002 | 6.99E-11 |
| rs35408430 | T | C | -0.022 | 0.342 | 0.002 | 1.72E-25 |
| rs36037728 | T | C | -0.058 | 0.026 | 0.006 | 2.50E-21 |
| rs3745669 | C | T | -0.013 | 0.372 | 0.002 | 3.19E-10 |
| rs3814995 | T | C | -0.013 | 0.312 | 0.002 | 3.06E-09 |
| rs3890624 | G | A | 0.012 | 0.395 | 0.002 | 1.32E-08 |
| rs4121823 | A | T | -0.019 | 0.845 | 0.003 | 1.20E-11 |
| rs41301394 | T | C | 0.012 | 0.282 | 0.002 | 3.19E-08 |
| rs4364259 | A | G | 0.016 | 0.202 | 0.002 | 2.88E-11 |
| rs4418728 | T | G | 0.012 | 0.452 | 0.002 | 1.05E-09 |
| rs4616820 | T | C | -0.012 | 0.465 | 0.002 | 5.30E-10 |
| rs512083 | C | T | 0.012 | 0.461 | 0.002 | 2.64E-09 |
| rs55707527 | T | G | 0.015 | 0.220 | 0.002 | 5.27E-10 |
| rs57459725 | G | C | -0.017 | 0.133 | 0.003 | 3.43E-09 |
| rs575976 | G | A | 0.016 | 0.182 | 0.003 | 1.06E-09 |
| rs5771043 | A | G | -0.012 | 0.328 | 0.002 | 3.46E-09 |
| rs58387006 | C | A | -0.014 | 0.222 | 0.002 | 1.09E-08 |
| rs6003465 | C | T | -0.012 | 0.332 | 0.002 | 7.12E-09 |
| rs61816766 | C | T | 0.087 | 0.029 | 0.006 | 3.05E-49 |
| rs62007299 | A | G | -0.012 | 0.713 | 0.002 | 4.20E-08 |
| rs62012775 | T | A | -0.016 | 0.176 | 0.003 | 5.79E-10 |
| rs62115743 | T | C | 0.027 | 0.082 | 0.004 | 1.24E-13 |
| rs62318873 | T | C | 0.054 | 0.047 | 0.005 | 1.93E-30 |
| rs6438900 | G | C | 0.013 | 0.258 | 0.002 | 3.53E-09 |
| rs6547409 | T | C | 0.028 | 0.050 | 0.005 | 1.19E-09 |
| rs6671730 | A | G | -0.015 | 0.434 | 0.002 | 7.76E-14 |
| rs6672758 | T | C | 0.016 | 0.801 | 0.002 | 2.98E-11 |
| rs6782190 | A | G | -0.019 | 0.648 | 0.002 | 2.32E-20 |
| rs6834488 | T | C | -0.014 | 0.424 | 0.002 | 3.26E-13 |
| rs700065 | T | G | -0.018 | 0.861 | 0.003 | 6.60E-11 |
| rs7128011 | A | G | -0.075 | 0.368 | 0.002 | 1.00E-200 |
| rs71297391 | T | C | 0.025 | 0.048 | 0.005 | 4.84E-08 |
| rs727857 | A | G | -0.011 | 0.611 | 0.002 | 1.66E-08 |
| rs73413596 | C | T | 0.023 | 0.074 | 0.004 | 1.08E-09 |
| rs7528419 | G | A | 0.020 | 0.225 | 0.002 | 1.75E-17 |
| rs75765985 | A | G | 0.053 | 0.016 | 0.008 | 9.22E-12 |
| rs77037130 | A | G | -0.067 | 0.015 | 0.008 | 4.06E-17 |
| rs77924615 | A | G | -0.015 | 0.193 | 0.003 | 1.28E-09 |
| rs78649910 | A | T | -0.020 | 0.106 | 0.003 | 5.61E-10 |
| rs8018720 | C | G | -0.038 | 0.823 | 0.003 | 2.11E-49 |
| rs80204526 | A | C | -0.053 | 0.011 | 0.010 | 3.91E-08 |
| rs804281 | G | A | 0.015 | 0.584 | 0.002 | 1.75E-14 |
| rs8063565 | C | G | 0.013 | 0.734 | 0.002 | 3.65E-09 |
| rs8091117 | A | C | -0.026 | 0.065 | 0.004 | 1.09E-10 |
| rs8107974 | T | A | 0.039 | 0.076 | 0.004 | 1.84E-25 |
| rs8121940 | G | C | -0.038 | 0.195 | 0.002 | 1.88E-52 |
| rs8123293 | G | A | 0.028 | 0.113 | 0.003 | 5.19E-20 |
| rs9476310 | T | C | 0.012 | 0.511 | 0.002 | 3.45E-09 |
| rs9490317 | C | T | 0.011 | 0.446 | 0.002 | 2.46E-08 |
| rs964184 | C | G | 0.043 | 0.868 | 0.003 | 2.03E-50 |
| rs9861009 | C | T | 0.015 | 0.728 | 0.002 | 3.57E-11 |

**Table S2** Details of the instrument variables for circulating 25(OH)D concentration included in MR analysis.

| **SNP** | **effect_allele** | **other_allele** | **eaf** | **beta** | **se** | **pval** |
| --- | --- | --- | --- | --- | --- | --- |
| rs3755967 | T | C | 0.28 | -0.089 | 0.002 | 4.74E-343 |
| rs12785878 | T | G | 0.75 | 0.036 | 0.002 | 3.80E-62 |
| rs10741657 | A | G | 0.4 | 0.031 | 0.002 | 2.05E-46 |
| rs17216707 | T | C | 0.79 | 0.026 | 0.003 | 8.14E-23 |
| rs10745742 | T | C | 0.4 | 0.017 | 0.002 | 1.88E-14 |
| rs8018720 | C | G | 0.82 | -0.017 | 0.003 | 4.72E-09 |

**Table S3** Information about GWAS utilized in the current study.

| **Trait** | **Ancestry** | **Consortium** | **Cases** | **Control** | **Gwas ID** | **Gender** |
| --- | --- | --- | --- | --- | --- | --- |
| Serum 25(OH)D levels | European | IEU consortium | 417580 | | ebi-a-GCST90000616 | Both sex |
| Circulating 25(OH)D concentration | European | 31 cohorts | 79366 | | NA | Both sex |
| Low back pain | European | FinnGen Consortium | 21140 | 227388 | NA | Both sex |
| Low back pain experienced last month | European | IEU consortium | 118471 | 343386 | [ukb-b-9838](https://gwas.mrcieu.ac.uk/datasets/ukb-b-9838/) | Both sex |
| Alcohol intake frequency | European | IEU consortium | 462346 | | [ukb-b-5779](https://gwas.mrcieu.ac.uk/datasets/ukb-b-5779/) | Both sex |
| BMI | European | IEU consortium | 461460 | | [ukb-b-19953](https://gwas.mrcieu.ac.uk/datasets/ukb-b-19953/) | Both sex |
| obesity | European | IEU consortium | 4688 | 458322 | **ukb-b-15541** | Both sex |
| Vitamin D deficiency | European | FinnGen Consortium | 303 | 292997 | NA | Both sex |
